# Tumour angiogenesis as a chemo-mechanical surface instability

**DOI:** 10.1038/srep22610

**Published:** 2016-03-07

**Authors:** Chiara Giverso, Pasquale Ciarletta

**Affiliations:** 1 Dipartimento di Matematica - MOX, Politecnico di Milano and Fondazione CEN, Piazza Leonardo da Vinci, 32-20133 Milano, Italy; 2 CNRS and Sorbonne Universités, UPMC Univ Paris 06, UMR 7190, Institut Jean le Rond d’Alembert, 4 place Jussieu case 162, 75005 Paris, France

## Abstract

The hypoxic conditions within avascular solid tumours may trigger the secretion of chemical factors, which diffuse to the nearby vasculature and promote the formation of new vessels eventually joining the tumour. Mathematical models of this process, known as tumour angiogenesis, have mainly investigated the formation of the new capillary networks using reaction-diffusion equations. Since angiogenesis involves the growth dynamics of the endothelial cells sprouting, we propose in this work an alternative mechanistic approach, developing a surface growth model for studying capillary formation and network dynamics. The model takes into account the proliferation of endothelial cells on the pre-existing capillary surface, coupled with the bulk diffusion of the vascular endothelial growth factor (VEGF). The thermo-dynamical consistency is imposed by means of interfacial and bulk balance laws. Finite element simulations show that both the morphology and the dynamics of the sprouting vessels are controlled by the bulk diffusion of VEGF and the chemo-mechanical and geometric properties at the capillary interface. Similarly to dendritic growth processes, we suggest that the emergence of tree-like vessel structures during tumour angiogenesis may result from the free boundary instability driven by competition between chemical and mechanical phenomena occurring at different length-scales.

Angiogenesis is the complex process by which new blood vessels develop from an existing vasculature in order to supply nutrients and/or metabolites to tissues, playing a fundamental role in many physiological and pathological conditions^1^,^2^,^3^,^4^,^5^.

For instance, angiogenesis physiologically occurs during embryogenesis, placenta formation, fetal development^6^ and during tissue-repair^7^. On the other hand, it may drive the onset and the progression of rheumatoid disease^8^, some eye diseases, duodenal ulcers and the initiation and growth of most types of solid tumors^9^,^10^.

Since angiogenesis involves the dynamics of the endothelial cells (ECs) forming the blood vessel wall, we investigate whether it is possible to develop a thermodynamically consistent *surface growth model* describing the onset of new vessels, unlike existing mathematical approaches requiring some empirical rules for capillary formation.

Angiogenesis invariably occurs through a well-ordered sequence of events driven by the rearrangement and migration of ECs (i.e. sprouting) forming the lining of the existing vasculature, and the subsequent EC proliferation and fusion, culminating in the formation of a network of new capillaries^11^,^12^.

In this paper, we focus on tumour-induced angiogenesis, the process in which a small avascular tumour (i.e. a colony of cancer cells that lacks its own blood supply) reaches a critical diameter of approximately 2 mm. Above this critical size, the existing vasculature can no longer sustain tumor growth only by means of nutrients and oxygen diffusion^9^. Tumour cell hypoxia is assumed to trigger the first event of tumor-induced angiogenesis, which is the secretion by tumor cells of a number of chemicals^5^, collectively called tumour angiogenic factors (TAFs)^13^. These substances diffuse through the surrounding tissue until they reach the nearby vasculature, whereupon they initiate the degradation of the basal membrane of the capillary wall^5^. Then, ECs respond to the TAF concentration gradient by proliferating near the sprout tip and by chemotactically migrating towards the TAF source, forming protrusions (sprouts)^14^,^15^,^16^. Following the initial small finger-like protrusion, branching later occurs (i.e. sprout branching)^4^,^14^,^17^. The growing branches move towards the tumour, following the motion of the leading EC at the sprout-tip and organize themselves into sort of a dendritic structure^4^,^15^,^16^,^17^ inside the extracellular matrix (ECM). Furthermore, as the sprout approaches the tumor, eventually joining the tumour mass, the branches dramatically increase in number^18^. Once the tumour has granted access to the vasculature, it gains access to a virtually endless supply of nutrients, possibly metastasizing in distant sites^1^,^4^.

Tumor angiogenesis is an active field of research not only for the biologists but also for the physical and mathematical researchers. In particular, several continuous^19^,^20^,^21^,^22^ and hybrid^23^,^24^,^25^,^26^ models have been proposed in the past 20 years^27^,^28^,^29^. These works focused mainly on the role played by ECs and by the different chemicals, including both those associated with the soluble angiogenic factors secreted by the cancer cells, and eventually those arising from insoluble molecules in the ECM^2^. The modelling of the interactions between the ECs and both the different angiogenic factors and ECM macromolecules typically encapsulates systems of coupled nonlinear partial differential equations (PDEs) describing the migration of ECs from the parent vessel towards the solid tumour. Such PDEs continuous models, first proposed by Balding and McElwain^20^ and later refined^4^, can describe some important features of angiogenesis occurring at the macroscopic scale, such as average sprout density, average vessel growth rates and network expansion rates^17^. However, they are not able to reproduce the morphology of the developing capillary network, since they use a diffuse interface approach. Therefore this kind of model can be used neither to capture the overall dendritic structure of the network nor to evaluate the inner blood flow^24^. The finer description of these vessel networks can be reproduced with some discrete models that operate at the scale of a single EC^17^,^30^,^31^. Thus some hybrid models combining a continuous representation for the chemicals and discrete elements to track the motion of individual ECs have been proposed^3^,^4^,^23^,^25^,^26^,^32^,^33^. Although discrete/hybrid models have the advantage of describing the motion of individual ECs for simulating a realistic capillary network, empirical rules for branching should be generally defined. Moreover, a proper mechanical description of the interactions occurring between the ECs and the ECM has not be incorporated^23^.

Furthermore, both the continuous and the discrete mathematical models in the state-of-the-art focused uniquely on the formation of the capillary sprout network in response to soluble and unsoluble chemical stimuli (e.g. TAFs and eventually fibronectin), without giving a proper mechanical representation of the process. Notwithstanding, in addition to chemical stimuli, mechanical cues play a fundamental role in vascular sprouting and maturation, since they govern the interaction between ECs and the surrounding extracellular environment^4^. Indeed, ECs interact with the ECM components, which strongly affect the cellular migration characteristics^4^,^19^,^22^,^34^. An extensive description of the key chemical and mechanical processes occurring during angiogenesis can be found in recent experimental works^35^,^36^,^37^.

In this work, we propose an original mechanistic approach to angiogenesis, defining a thermo-dynamically-consistent continuous model of interfacial growth, that takes into account geometrical, physical and chemo-mechanical factors.

Inspired by the striking similarity to tree-like structures found in pure liquid solidification, isothermal solidification of liquid mixtures and oil recovery by fluid injection^38^,^39^, we resort to the theory of interfacial growth^40^ and pattern formation in crystal growth^39^,^41^, proposing a continuous model with sharp interface. These theories have largely been applied to inert matter, ranging from the growth of snowflakes to the solidification of metals^38^, and recently employed for living systems, such as bacterial colonies^42^,^43^, cell differentiation during morphogenesis and cancer growth^44^. Compared to non-living systems, biological processes present a significantly higher complexity in the mechanisms of self-organization, even if some general principles can be still applied^41^,^42^. All these processes, indeed, are characterized by the occurrence of an interfacial pattern, i.e. the developing structures will form at the interface between two (meta-) stable phases/materials rather than in the bulk of the material. Furthermore, complex patterns result from out-of-equilibrium phenomena dominated by the interplay of driving forces of different physical nature^38^,^39^. Our understanding of this mechanism is given by the Mulins-Sekerka instability^41^,^45^, which describes pattern formation during the solidification of a solid phase front growing into a supercooled liquid^39^.

In the following we propose a free-boundary mathematical model of tumor-induced angiogenesis. The model encapsulates the interfacial and bulk balance laws for ECs and extracellular space, respectively, as well as the reaction-diffusion equation for the vascular endothelial growth factor (VEGF), one of the principal tumour angiogenic factors^15^,^26^. Implementing a finite element code, we later present the numerical simulations of the model, together with a sensitivity analysis on the model parameters, which highlights the key factors controlling the vascular morphology. Finally, we critically discuss the numerical results of the proposed model and we add few concluding remarks.

## Mathematical Model

### Description of the biological system model

Solid tumors can stimulate new vessel formation^10^ across distances of some millimeters (from 1–3 mm^46^ to 5 mm^47^), whilst it takes approximately 10–21 days for the growing network to link the tumour to the parent vessel^14^,^18^,^47^. However, generally, mathematical models generally focus on smaller length-scales (e.g. 17–68 *μ*m^3^).

**Figure 1:**
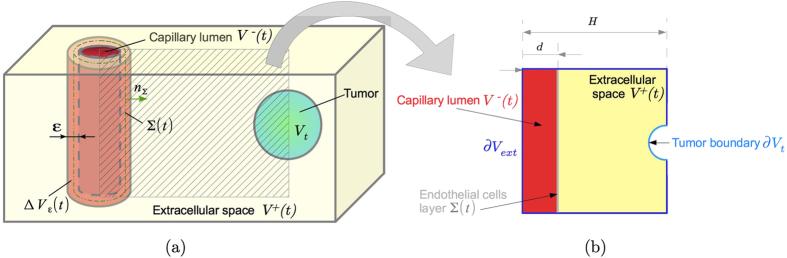
(**a**) Schematic representation of the biological domain consider: the capillary lumen, the extracellular space and the tumor can be represented through the control volumes *V*^−^ (*t*), *V*^+^ (*t*) and *V*_*t*_ (*t*), respectively, whereas the endothelial layer of the capillary is represented through the non-material interface, Σ(*t*). (**b**) 2D domain used for the numerical simulations, representing the section of the 3D domain in (**a**).

The major biological components of the vessel are ECs, forming a mono-layer of flattened and extended units. The abluminal surface of the capillary is then wrapped into the basal lamina, which is a collageneous network composed by laminin, other proteins and carbohydrates. The thickness of the basal lamina is only a fraction of the endothelial layer, so that the typical dimension of the capillary wall (including both the basal lamina and the endothelial layer) is in the order of 190–270 nm, for a capillary lumen spanning from 4–5 *μ*m up to 30–40 *μ*m 48. Thus all the relevant biological processes occurring during angiogenesis take place within a region Δ*V*_*ε*_ whose characteristic width is much smaller than the capillary-tumor distance and the lumen size (see Fig. 1a).

### Definition of the mathematical model

Accordingly, let us consider a continuous system made by two different materials occupying the two adjacent regions, *V*^+^, representing the extracellular space, occupied by the ECM and healthy tissue, and *V*^−^ representing the capillary lumen filled with blood. In the following, we will refer to all properties related to the material in *V*^+^ and *V*^−^ with the superscripts + and −, respectively (see Fig. 1b). Let us consider angiogenesis as an *interfacial growth* process^40^,^44^, i.e. as the ensemble of phase transformation phenomena occurring between the two contiguous materials in a very narrow volume Δ*V*_*ε*_ across their interface (see Fig. 1):




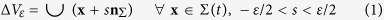




where **n**_Σ_ = **n**^−^ = −**n**^+^ is the local outward unit normal vector of the surface, *ε* is the small thickness of this interfacial volume and Σ(*t*) is the non-material interface that separates the two regions and that represents the capillary wall.

Thus, we can define the surface fields on the interface Σ(*t*) by homogenizing the volumetric physical properties defined inside the small volume Δ*V*_*ε*_^40^. In practice, the surface Σ(*t*) behaves as a moving non-material interface, which carries the thermo-chemo-mechanical properties of Δ*V*_*ε*_. Therefore Σ(*t*) can be treated as a discontinuity moving inside the continuous biological system with parametric velocity 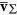 and associated physical velocity **v**_Σ_. Then we can assume^40^ that the projection **v**_Σ*s*_ on the surface Σ of the physical velocity **v** is uniform inside the volume Δ*V*_*ε*_, i.e.




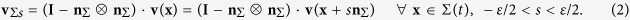




The tangential component 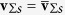 is dependent on the parametrization, whilst the normal component 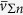 is not. Finally, we remark that the decomposition of the physical velocity is given by 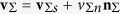, so that 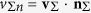 is unique.

In this framework, balance laws should be formulated not only for the volume fields but also for the surface fields, thus to guarantee the consistency of the thermo-chemo-mechanical properties of the whole system. Let *ρ*^*α*^, with *α* = {+, −}, be the spatial density in the corresponding volumes, we first derive the mass balance laws. Considering that no net proliferation occurs inside the volumes *V*^*α*^, with *α* = {+, −}, that the ECM degradation associated to matrix metalloproteinases is locally concentrated at the interface, the mass balance equation reads^40^,^44^




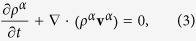




where we considered that non-convective mass fluxes are zero everywhere in the bulk volumes *V*^*α*^ but on both sides of the interface, possibly having a discontinuity across Σ(*t*). Since the spatial density *ρ*^*α*^ can be considered as constant, the impressibility condition in Eq. (3) is satisfied if we take **v**^*α*^ = 0, thus assuming that either the capillary sprout degrades and invades the extracellular space without deforming it^49^, or that the growing ECs fill *V*^−^ without inducing any volumetric deformation.

On the other hand, defining the homogenized surface density field *ρ*_Σ_ and surface mass source *γ*_Σ_, the mass balance of the surface density for the ECs of the capillary wall reads^40^,^44^




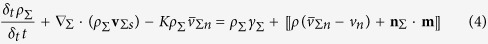




where ∇_Σ_ · (·) = (**I** − **n**_Σ ⊗_ **n**_Σ_) · ∇ is the surface divergence, *K* is twice the local mean curvature, *δ*_*t*_ indicates the Thomas (convected) derivative, 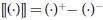 is the jump operator and ***m*** is a non-convective mass flux vector.

Taking *ρ*_Σ_ constant in time and in space, the mass balance for the surface density of ECs (5) leads to




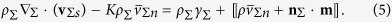




Inside the proliferative interface, new ECs are constantly produced by a surface mass source *γ*_Σ_, whereas the extracellular material is degraded^49^ in order to make the capillary advance and the waste material is transported inside the capillary in order to be eliminated. Therefore, we can define the mass fluxes **m**^*α*^ at the interface by setting




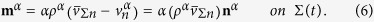




Assuming that *ρ*^+^ = *ρ*^*−*^, motivated by the fact that both materials are mainly composed by water, the previous relation states that the growing material deposited in the capillary lumen is created with the same rate of the degraded ECM at the interface. Substituting Eq. (6) into Eq. (5) and, for the sake of simplicity, restricting to surface divergence-free motion of the interface, we obtain that 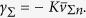 This interface balance law states that the proliferation of EC is proportional to the curvature, as observed in some biological experiments^50^.

For the sake of simplicity, we here consider only the effect of the VEGF, which is the main chemical involved in the early stage of angiogenesis^15^. In the proposed model, we assume that the secreted VEGF diffuses from the tumor cell located at the boundary ∂*V*_*t*_ (see Fig. 1) through the extracellular region and the intercapillary space, with the same diffusion coefficient *D*_*c*_, so that the balance of the VEGF concentration, *c*, reads




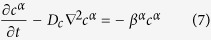




being *β*^*α*^ the decay rate of the substance in the volume *V*^*α*^. Since the VEGF is a diffusible factor with negligible inertia, the introduction of an interfacial field is not required and Eq. (7) must be complemented with the following boundary conditions




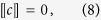







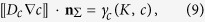




which represent the continuity of the chemical field and the jump of the normal gradient due to the absorption rate *γ*_*c*_(*K*,*c*) at the interface.

Let us now impose the balance of the physical linear momentum inside the biological system. Neglecting inertial terms and in absence of external forces, the balance of linear momentum inside the volume *V*^*α*^ reads




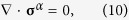




being ***σ***^*α*^ the Cauchy stress tensor of the material in the volume *V*^*α*^. As done for the mass balance, we have to consider also surface quantities along with volumetric ones. Therefore, defining the Cauchy stress tensor for the interface, ***σ***_Σ_, the balance of linear momentum for the interface, under the condition (6), reads




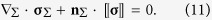




Considering that the living material in *V*^+^ and *V*^−^ behaves as a perfect fluid with surface tension ω, being **σ**_Σ_ = ω**I**_Σ_ = ω(**I** − **n**_Σ _⊗ **n**_Σ_), eq. (11) reduces to the standard Young-Laplace equation.

In order to impose the thermodynamical consistency of the model, the reduced dissipation inequalities inside the volume *V*^*α*^ and on the surface Σ(*t*) should be defined (see the Supplementary Information for further details). Since the VEGF concentration is continuous at the interface, the associated volumetric chemical potentials *μ*^*α*^ should also be continuous, i.e. 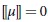. Accordingly, the quasi-static reduced dissipation inequality on Σ(*t*) (see the Supplementary Information) reads




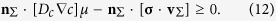




Under the assumption **v**_Σ*s*_ = 0, the substitution of (9) and (11) into (12) leads to γ_*c*_*μ* + *K*ω*v*_Σ*n*_ ≥ 0. In particular in the following we will consider the thermodynamically admissible condition




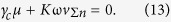




Furthermore, we will assume that the VEGF activates ECs in a region of a nearby capillary where the concentrations of the tumor angiogenic growth factor reaches a given threshold^4^,^35^, namely *c*_0_. Thus, the consumption term *γ*_*c*_ takes the following form




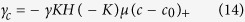




where *H* is the Heaviside function (i.e. *H*(−*K*) = 1 if *K* ≤ 0, else *H*(−*K*) = 0), (·)_+_ is the positive part of its argument and *γ* is the uptake of chemical energy at the interface, that in this work we assume to be a constant. The previous constitutive assumption allows rewriting eq. (13) as




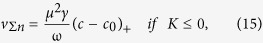




which allows deriving the normal velocity of the capillary wall. We remark that for *K* = 0 the relation (13) is automatically satisfied, so that *v*_Σ*n*_ has been extended for continuity also for *K* = 0. The normal velocity depends on the curvature of the interface, since the latest influences the chemical potential at the interface, being




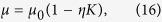




where *μ*_0_ is the nominal VEGF chemical potential and *η* is a characteristic microscopic length, in accordance with the Gibbs-Thompson condition^51^.

In summary, the mathematical model is given by eq. (7), with boundary conditions (8–9) on the moving discontinuity complemented by the absorption term (14), a fixed concentration *c*_*t*_ on the boundary of the tumor and no-flux conditions on the remaining border of the domain, i.e.




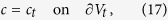







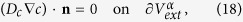




whereas the interface Σ(*t*) moves with normal velocity




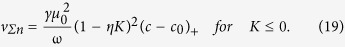




In order to perform some numerical simulations, it is useful to derive the non-dimensional system of governing equations and to obtain the fundamental parameters of the model. Considering the following characteristic time *t*_*c*_, length *l*_*c*_, velocity *v*_*c*_ and chemical concentration *c*_*c*_:




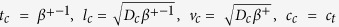




and using the barred notation to denote dimensionless quantities, the system to be solved reads




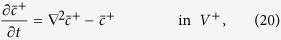







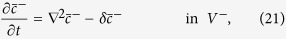







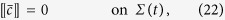







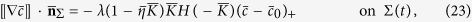







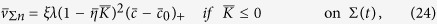







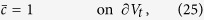







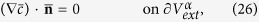




where *δ* = *β*^−^/*β*^+^, 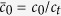, *λ* *=* *γμ*_0_*/D*_*c*_, 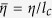, 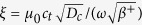.

The nondimensionalization procedure leads to the definition of five dimensionless parameters, *δ*, 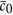, *λ*, 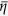, *ξ*. In particular:

- *δ* represents the ratio between the decay of VEGF inside and outside the vessel.
- *λ* is related to the ratio between the rate of absorption and the rate of diffusion of VEGF.
- *ξ* represents the ratio between chemical and mechanical energies driving capillary growth.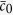 is merely the ratio between the threshold required for EC activation and the VEGF concentration at the tumor border.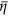 is the ratio between the microscopic length regulating chemical absorption at the interface and the VEGF diffusive length.

Finally the size characterizing the domain of the simulations are made dimensionless with respect to the characteristic length *l*_*c*_.

For sake of simplicity, in the following we will omit the barred notation to denote dimensionless quantities.

## Results

### Finite Element Simulations

The system of eqs (20, 21, 22, 23, 24, 25, 26) has been numerically implemented developing a finite element code with the open-source program FreeFem++^52^. The jump condition (23) at the moving boundary has been introduced in the variational formulation of the problem. The equations for the chemical species (20–21) are solved on a triangular grid, fitting at every iteration the moving interface. Given the VEGF concentration *c*_*i*_ at time *t*_*i*_, the curvature of the boundary *K*_*i*_ and the value of the chemical potential *μ*_*i*_, the normal velocity of the boundary *v*_Σ*n*,*i*_ is computed from eq. (24). The nodes belonging to the interface are then explicitly moved accordingly to the computed velocity in order to obtain the new position of the discontinuity at time *t*_*i*+1_. Once the new position is known, the curvature *K*_*i*+1_, and the chemical potential *μ*_*i*+1_ are updated in order to compute the chemical field at time *t*_*i*+1_, using an implicit-Euler scheme. Then, the mesh is adaptively refined, generating an increasing number of grid points in those region close to the higher curvature of the moving interface.

**Figure 2:**
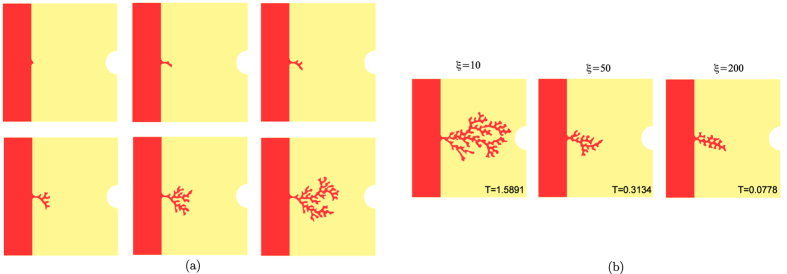
(**a**)Angiogenesis dynamics in numerical simulations: the initial sprout evolves and soon splits in second-generation vessels, which in turn split until a complex tree-like network is formed. The simulations have been obtained setting *ξ* = 10, *λ* = 1, *δ* = 500, *η* = 0.0316, *c*_0_ = 0.143 in a domain with dimensionless length and height equal to 0.316 and the interface initially placed at *x*_Σ_ = 0.079. The total dimensionless time of this simulation is *T* = 1.558. (**b**) Morphological diagram of the simulated capillary morphology for different values of the parameter *ξ*. The simulations were obtained setting *λ* = 1, *δ* = 100, *η* = 0.0316, *c*_0_ = 0.3432. We report at the bottom the dimensionless time at which each snapshot has been taken. The simulations stopped because of either the capillaries reached the tumour (left snapshot) or they formed anastomosis (central and right snapshots).

The spatio-temporal dynamics for the vessel formation resulting from numerical simulations is reported in Fig. 2a. Initially, a sprout arises from the parent vessel and it soon splits in two or more branches. Some of this second-generation branches do not grow sufficiently to give rise to third-generation branches (because the concentration of VEGF at their tips is not high enough), whereas some others soon split. The process continues until a dendritic structure is formed and the solid tumor is reached. Accordingly to biological observations^14^,^18^,^47^, the branching velocity increases as the vessels approach the tumor, due to the increase in the VEGF concentration close to the tumour region and the formation of an increased number of branches with higher absolute curvatures. The visible higher frequency of branching at the edge of the network as the capillary sprouts approach the tumor is biologically known as the “brush border” effect^14^,^18^.

### Sensitivity analysis

The growth of the network evolves both temporally and spatially in response to the combined effects of angiogenic factors, migratory cues via the ECM, mechanical factors acting at the vessel interface (i.e. the surface tension) and geometric factors, that are summarized in the model dimensionless parameters. Therefore, we conducted a sensitivity analysis changing one dimensionless parameter at a time, while keeping the other parameters fixed. In particular, Fig. 2b reports the final simulated morphologies for different values of the parameter *ξ*, that drives, along with *λ*, the velocity of the network. We find that the vessel sprouting is strongly favored by smaller values of the parameter *ξ*, i.e. by low velocities, which is a common feature of growing biological systems in a diffusion-limited regime^41^. Since *ξ* is proportional to the ratio between the chemical energy of VEGF diffusion and the mechanical energy of the capillary tip (ω), small values of *ξ* correspond to situations in which the capillary surface tension is dominant on the diffusion of chemicals. In contrast to the patterns emerging in other non-living and living systems^39^,^42^,^43^, here the surface tension does not act as a stabilizing effect at small wavelengths, but it rather promotes the formation of lateral ramified branches. Indeed, the instability is governed in this problem by the curvature of the interface, similarly to dendritic growth problems^41^, and the only short-scale cutoff is the size of the ECs in the capillary wall.

**Figure 3:**
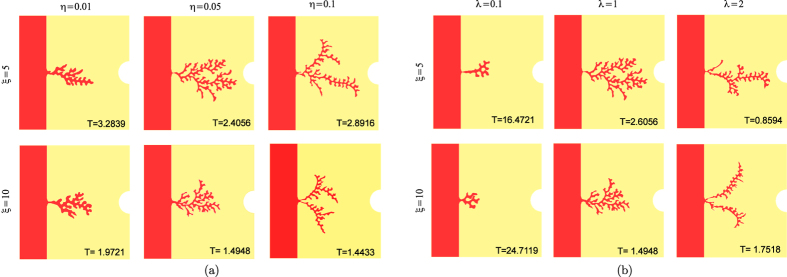
(**a**) Morphological diagram of the simulated capillary morphology for different values of the parameter *η* and *ξ*, setting *λ* = 1, *δ* = 100, *c*_0_ = 0.33838 in a domain with dimensionless length and height equal to 0.32 and the interface initially placed in *x*_Σ_ = 0.08. (**b**) Morphological diagram of the simulated capillary morphology for different values of the parameter *λ* and *ξ*, setting *δ* = 100, *η* = 0.05, *c*_0_ = 0.33838 in a domain with dimensionless length and height equal to 0.32 and the interface initially placed in *x*_Σ_ = 0.08. At the bottom of each snapshot we reported the dimensionless time at which it was taken.

The parameter *η* is also very influential for the formation of the capillary network, since it weights the increase of VEGF absorption, thus regulating the velocity of the front. The dimensionless *η* represents the ratio between the microscopic length related to the chemical absorption at the interface and the characteristic diffusive length inside the volumes. Figure 3a reports the different morphologies obtained varying the parameter *η* for different values of *ξ*, showing that more branched patterns with sharper tips arise for higher values of *η*. Indeed, the microscopic length related to the chemical dynamics at the interface is known to give the dimensional information to set the characteristic microscopic scale of a pattern in pure solidification^41^. Actually, smaller values of *η* significantly reduces the contribution of the curvature to the velocity, whereas higher values of this parameter amplify even small variations in the curvature, setting the formation of sharper tips.

Sharper and thinner vessel tips can be obtained also increasing the parameter *λ* (see Fig. 3b), which weights the absorption of VEGF at the vessel interface: since the absorption is proportional to the curvature, higher values of *λ* generates a sort of “tip-effect”, sequestering the VEGF on the tips of the vessel and thus hindering the formation of long later protrusion behind the front-head tip. For small values of *λ*, the finger-like capillary sprouts tend to bend toward each other, as observed in experiments^53^. This process will eventually lead to numerous tip-to-tip and tip-to-sprout fusions known as anastomoses^15^. In the present model, we do not implement the formation of anastomoses, for increased technical complications, and the simulations stops as soon as two branches touch.

The effect of varying the parameters *β*, which is the ratio between the VEGF decay inside the vessel and inside the extracellular environment, strongly influence the evolution of the chemical field and the initial condition on the VEGF (obtained solving the stationary problem, without absorption on the vessel wall). Therefore in order to obtain the same initial thickness of the initial sprout, we have to change the initial value of *c*_0_: in particular, as *δ* increases we have to set a smaller threshold *c*_0_ in order to observe the formation of branches. In all cases, the time required to form the branched vessel network decreases with increasing *δ*.

**Figure 4:**
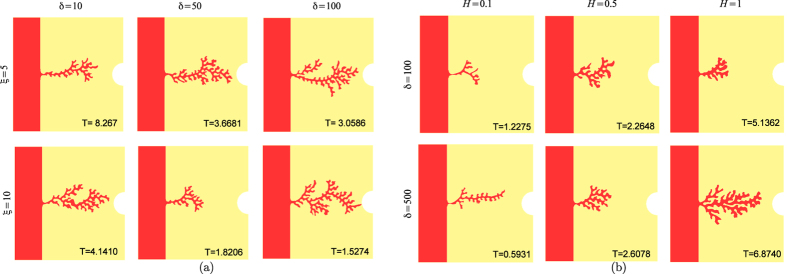
(**a**) Morphological diagram of the simulated capillary network for different values of the parameter *δ* and *ξ*, setting *λ* = 1, *η* = 0.032 in a domain with dimensionless length and height equal to 0.32 and the interface initially placed in *x*_Σ_ = 0.08. The value of *c*_0_ changes in order to keep constant the initial sprout size. (**b**) Morphological diagram of the simulated capillary morphology for different dimension of the domain of simulation (*H*), setting *λ* = 1, *η* = 0.032 and *ξ* = 10. The interface is placed at distance *H*/4 from the left-side of the domain and *c*_0_ is chosen in order to keep constant the ratio between the initial sprout size and the domain dimension *H*. At the bottom of each snapshot we reported the dimensionless time at which it was taken.

Finally, we consider the effect of varying the size of the domain, keeping constant the ratio between the size of the capillary, *d*, and the the total size of the square domain, *H* (see Fig. 1b). Figure 4 shows that relatively thicker branches (with respect to the normalized initial sprout thickness) can be obtained as *H* increases, highlighting the existence of a size effect in the pattern selection.

### Physical interpretation of the model results

The tree-like network structure emerging in the numerical simulations is somewhat similar to the morphology of crystal fronts in solidification problems, where a stable thermodynamic phase propagates into a metastable one. In both cases, the competition is between the diffusion of a given field (e.g. thermal or chemical) on one hand, and the microscopic dynamics occurring at the interface (such as the surface tension and the chemical kinetics of absorption at the interface) on the other, modulated by the ratio between the typical distances involved in the process and the diffusive length. The result of this competition is the onset of branches and the formation at the macroscopic scale of intricate tree-like structures, which closely resemble to the ones experimentally observed^54^,^55^. Thus, this work shows that the onset of vessel sprouting from the existing vasculature can be reproduced without defining ad-hoc empirical rules for branching, as in previous mathematical approaches, but it simply results from the interactions between chemical and mechanical phenomena, generating an unstable growth process at the vessel interface.

Unfortunately, a direct quantitative comparison between the numerical simulations and the biological experiments is not straightforward, since not all the data required by the mathematical model, even though measurable in principle, are reported in literature.

However, it is useful to compare our results with the available experimental data with respect to the size of the domain and the time required for forming the vascular network. The diffusion coefficient inferred from biological data, for the vast majority of angiogenic growth factors, is in the order of 10–600 *μ*m^2^/s 4,56,57, while the decay rate of the VEGF in the surrounding tissue is in the order of 0.456–0.65 h^−1^ 56,58. Accordingly, taking *D*_*c*_ = 10 *μ*m^2^/s would results in a computational domain having a width of about 90μm, which is comparable to the value used in previous mathematical models^3^; whereas *D*_*c*_ = 600 *μ*m^2^/s would represent a width of about 0.7 mm, that is closer to the tumor-capillary distance reported in literature^5^,^58^. For what concern the dynamics, experiment on the avascular cornea have shown that angiogenetic processes take 2–5 days to form an initial sprout^59^. Once the sprouts are developed, the growth rate of new capillaries is much faster, with an initial growth rate of about 0.22 mm/day which increases up to 0.61 mm/day when the network becomes highly branched^59^. An increasing velocity over time has also been reported in previous mathematical models^3^, precisely from ~0.20 mm/day to ~1 mm/day, highlighting the long time required for the initial spouting. These effects are consistent with our numerical results, where the initial sprout formation requires a long time, whereas branching accelerates as the new capillaries approach the tumor (see the Supplementary Information for further details). Even if our results at this stage do not have any intent of being quantitative, the proposed model should be regarded as a proof-of-concept to outline a new mechanism of angiogenesis formation.

## Discussion

In this work we have presented a theoretical and numerical analysis for studying the well-orchestrated sequence of biological events occurring during tumour angiogenesis. In particular, we have proposed a thermodynamically-consistent growth model describing the morphology of the new developing vasculature, depending on the different geometrical and chemo-mechanical factors involved in the initial stages of network formation. The proposed continuous approach couples the diffusion of angiogenic molecules (VEGF) with the chemically-driven migration and proliferation of ECs. Unlike existing modelling approaches^19^,^20^,^21^,^22^ we explicitly reproduce both the temporal and spatial network evolution taking into account for the chemo-mechanical cues (i.e. the surface tension, the variation of the chemical potential across a curved interface) and geometric factors (i.e. the capillary to tumour distance). In addition, this application of a mechanistic interface growth model to angiogenesis is completely new. Our approach brings novel insights on the role played by physical forces along with chemical factors in directing angiogenesis. In particular, five dimensionless parameters, encapsulating the geometric, chemical and mechanical cues, are found to characterize the model dynamics: *δ*, 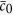, *λ*, *η*, *ξ*. The effects of the different parameters on the predicted morphology are studied in the numerical simulations. Angiogenesis initiates with a first vessel tip sprouting when the local VEFG concentration reaches a given threshold. Such a protrusion undergoes an unstable growth process, with the curvature having a destabilizing effect on the capillary motion. Indeed the simulations demonstrate that the both the microscopic chemical kinetics at the interface of the capillary, following the Gibbs-Thompson law, and the size effects of the biological domains compete with the reaction-diffusion of VEGF in order to determine the occurrence of complex branched patterns. The predicted morphologies resulting from the numerical simulations strikingly resemble the tree-like vascular structures experimentally observed *in vivo* and *in vitro*^54^,^55^. Nonetheless, this work should be regarded as a proof-of-concept of the fundamental role of physical forces during angiogenesis, since it neglects some molecular processes acting at the cellular level. Thus, future studies should focus on the incorporation of the molecular and other cellular mechanisms observed in biological experiments (e.g. the Notch signaling^16^,^30^,^60^, the interaction between stalk cell and tip cell^16^,^60^). Moreover, we remark that in this simulations the concentration of VEGF is kept constant at the tumor boundary, whereas time-dependent conditions at the tumor interface can be useful to consider the physiological feedback between the tumor oxygenation due to the onset of the new vasculature and the secretion of VEGF in response to hypoxic condition. Taking this feedback mechanism into accoun might affect the dynamics of vessel formation as the branches approaches the tumor. It is also worth noticing that the proposed numerical implementations should be refined in order to describe the formation of anastomosis and the role played by the environmental stress in influencing capillary branching. In fact, despite the mathematical model has been derived considering the role of the stress field in the surrounding environment, the simulations have been performed considering an external inviscid fluid and a surface tension acting at the interface. By formulating proper hypotheses on the mechanical stress exerted by the surrounding tissue, future works should focus on the role played by external mechanical cues on the overall process. Indeed, there is a growing recognition that the balance between internally generated and externally applied forces, along with ECM remodelling and mechanical factors connected with blood flow or extravascular mechanical stress are key determinants of a cell’s fate and function and are important regulators in postnatal physiological angiogenesis^61^,^62^,^63^,^64^,^65^. Nevertheless, mature ECs can develop capillary-like networks in cell culture even in the absence of flow or any other externally applied stresses^61^, demonstrating that extrinsic stresses are not strictly necessary for triggering angiogenesis. Furthermore, even if the majority of the existing mathematical models of angiogenesis are 2D^1^,^3^,^4^,^25^, angiogenesis is typically a 3D process, with tips sprouting in directions other than that of the propagating vascular front. Thus, future works will certainly focus on the 3D implementation of the proposed model. In spite of these limitations, the model provides original insights about the influence of the physical and chemical effects on the pattern dynamics during angiogenesis. In conclusion, the proposed mechanistic approach, possibly combined with biologically more detailed diffusion-based mathematical models, has the potential to foster our understanding on the process of vessel formation. A deeper comprehension of the key factors directing angiogenesis is fundamental for many clinical applications, since vessel tortuosity is know to strongly affect the anti-tumor treatment response^66^,^67^. Finally, even though we focus exclusively on tumour-induced angiogenesis, the proposed model can be useful to model other biological processes, such as wound healing^68^ and tissue optimization in engineering scaffolds^69^.

## Additional Information

**How to cite this article**: Giverso, C. and Ciarletta, P. Tumour angiogenesis as a chemo-mechanical surface instability. *Sci. Rep.* **6**, 22610; doi: 10.1038/srep22610 (2016).

## Supplementary Material

Supplementary Information
