# Erratum: Tumour angiogenesis as a chemo-mechanical surface instability

**DOI:** 10.1038/srep25214

**Published:** 2016-04-29

**Authors:** Chiara Giverso, Pasquale Ciarletta

Scientific Reports 6: Article number: 22610; 10.1038/srep22610 published online: 03072016; updated: 04292016.

This Article contains typographical errors.

In Equation 12,


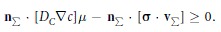


should read:


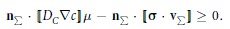


In the Acknowledgements section,

“This work is funded by the AIRC MFAG grant 17412, partially supported by partially supported by the “Start-up Packages and PhD Program” project”.

should read:

“This work is funded by the AIRC MFAG grant 17412, partially supported by the “Start-up Packages and PhD Program” project”.

