# Supplementary material for: Tumour angiogenesis as a chemo-mechanical surface instability
Source: Sci Rep. 2016 Mar 7;6:22610. doi: 10.1038/srep22610 (PMC4780075; doi:10.1038/srep22610)
Supplement: Supplementary Information [file srep22610-s1.pdf]

Tumour angiogenesis as a chemo-mechanical surface instability

Supplementary Information

Chiara Giverso [1], Pasquale Ciarletta [2]

1 Theoretical Derivation

In order to close the description of the process in a thermodynamically consistent way, we have to consider the balance of the internal energy. Neglecting kinetic terms, calling ϵ^α the internal energies per unit mass in the volumes and ϵ_Σ the internal energies per unit mass on the surface, the local forms of the internal energy conservation read

$$\begin{aligned}\frac{\partial \rho^\alpha \epsilon^\alpha}{\partial t} + \nabla \cdot (\rho^\alpha \epsilon^\alpha \mathbf{v}^\alpha) &= \rho^\alpha \gamma^\alpha \epsilon^\alpha - \beta^\alpha c^\alpha \mu^\alpha + \nabla \cdot (\mathbf{m}^\alpha \epsilon^\alpha) + \\ &+ \nabla \cdot (D_c^\alpha \nabla c^\alpha \mu^\alpha) + \mathbf{T}^\alpha : \nabla \mathbf{v}^\alpha \end{aligned} \tag{SI.1}$$

$$\begin{aligned}\frac{\delta_t \rho_\Sigma \epsilon_\Sigma}{\delta_t t} + \nabla_\Sigma \cdot (\rho_\Sigma \epsilon_\Sigma \mathbf{v}_{\Sigma s}) &= K \rho_\Sigma \epsilon_\Sigma \bar{v}_{\Sigma n} + \rho_\Sigma \gamma_\Sigma \epsilon_\Sigma + \mathbf{T}_\Sigma : \nabla_\Sigma \mathbf{v}_\Sigma + \\ + [\![\rho \epsilon (\bar{v}_{\Sigma n} - v_n) &+ \mathbf{n}_\Sigma \cdot (\mathbf{m}\epsilon + \mathbf{T} \cdot (\mathbf{v} - \mathbf{v}_\Sigma) + D_c \nabla c \mu)]\!] \end{aligned} \tag{SI.2}$$

where μ^α is the volumetric chemical potential associated to the VEGF factor inside the region V^α. Then, the local entropy inequalities on the volume V^α and on the surface Σ takes the forms

$$\frac{\partial \rho^\alpha \eta^\alpha}{\partial t} + \nabla \cdot (\rho^\alpha \eta^\alpha \mathbf{v}^\alpha) \geq \rho^\alpha \gamma^\alpha \eta^\alpha + \nabla \cdot (\mathbf{m}^\alpha \eta^\alpha) \tag{SI.3}$$

$$\frac{\delta_t \rho_\Sigma \eta_\Sigma}{\delta_t t} + \nabla_\Sigma \cdot (\rho_\Sigma \eta_\Sigma \mathbf{v}_{\Sigma s}) \geq K \rho_\Sigma \eta_\Sigma \bar{v}_{\Sigma n} + [\![\rho \eta (\bar{v}_{\Sigma n} - v_n) + \mathbf{n}_\Sigma \cdot (\mathbf{m}\eta)]\!] \tag{SI.4}$$

where we called η^α the entropy associated to the volume V^α and η_Σ the entropy associated to the surface Σ.

[1] C. Giverso
Dipartimento di Matematica - MOX, Politecnico di Milano and Fondazione CEN
Piazza Leonardo da Vinci, 32 - 20133 Milano, Italy

[2] P. Ciarletta
Dipartimento di Matematica - MOX, Politecnico di Milano and Fondazione CEN
Piazza Leonardo da Vinci, 32 - 20133 Milano, Italy
CNRS and Sorbonne Universités, UPMC Univ Paris 06, UMR 7190, Institut Jean le Rond d'Alembert,
4 place Jussieu case 162, 75005 Paris, France
E-mail: pasquale.ciarletta@polimi.it

Considering the mass conservation equations , the equations for the internal energy (SI.1) - (SI.2) and the ones for the entropy (SI.3) -(SI.4) simplify in

$$\begin{aligned} \rho^\alpha \dot{\epsilon}^\alpha &= \mathbf{m}^\alpha \cdot \nabla \epsilon^\alpha + \mathbf{T}^\alpha : \nabla \mathbf{v}^\alpha - \beta^\alpha c^\alpha \mu^\alpha + \nabla \cdot (D_c^\alpha \nabla c^\alpha \mu^\alpha) && \text{(SI.5)} \\ \rho_\Sigma \dot{\epsilon}_\Sigma &= \mathbf{T}_\Sigma : \nabla_\Sigma \mathbf{v}_\Sigma + [\![(\epsilon - \epsilon_\Sigma)(\rho(\bar{v}_{\Sigma n} - v_n) + \mathbf{n}_\Sigma \cdot \mathbf{m}\epsilon)]\!] \\ &+ \mathbf{n}_\Sigma \cdot [\![\mathbf{T} \cdot (\mathbf{v} - \mathbf{v}_\Sigma) + D_c \nabla c \mu]\!] && \text{(SI.6)} \\ \rho^\alpha \dot{\eta}^\alpha &\geq \mathbf{m}^\alpha \cdot \nabla \eta^\alpha && \text{(SI.7)} \\ \rho_\Sigma \dot{\eta}_\Sigma &\geq [\![(\eta - \eta_\Sigma)(\rho(\bar{v}_{\Sigma n} - v_n) + \mathbf{n}_\Sigma \cdot \mathbf{m})]\!] \,. && \text{(SI.8)} \end{aligned}$$

where $\dot{\epsilon}^\alpha = \dfrac{\partial \epsilon^\alpha}{\partial t} + \mathbf{v}^\alpha \cdot \nabla \epsilon^\alpha$ and $\dot{\epsilon}_\Sigma = \dfrac{\delta_t \epsilon_\Sigma}{\delta_t t} + \mathbf{v}_{\Sigma s} \cdot \nabla_\Sigma \epsilon_\Sigma$.

Finally, assuming that the temperature field T is continuous across the interface and defining the Helmholtz free energy per unit of mass inside the volume $\Psi^\alpha = \epsilon^\alpha - T\eta^\alpha$ and the Helmholtz free energy per unit mass of the surface $\Psi_\Sigma = \epsilon_\Sigma - T\eta_\sigma$, it is possible to obtain the reduced dissipation inequalities for the growing system subtracting either Eq. (SI.7) or (SI.8) multiplied by T from Eq. (SI.5) or Eq. (SI.6), respectively,

$$\begin{aligned} \rho^\alpha \dot{\Psi}^\alpha &\leq -\beta^\alpha c^\alpha \mu^\alpha + \nabla \cdot (D_c^\alpha \nabla c^\alpha \mu^\alpha) + \\ &+ \mathbf{m}^\alpha \cdot (\nabla \Psi^\alpha + \eta^\alpha \nabla T) + \mathbf{T}^\alpha : \nabla \mathbf{v}^\alpha && \text{(SI.9)} \\ \rho_\Sigma \dot{\Psi}_\Sigma &\leq \mathbf{T}_\Sigma : \nabla_\Sigma \mathbf{v}_\Sigma + [\![(\Psi - \Psi_\epsilon)(\rho(\bar{v}_{\Sigma n} - v_n) + \mathbf{n}_\Sigma \cdot \mathbf{m})]\!] + \\ &+ \mathbf{n}_\Sigma \cdot [\![\mathbf{T} \cdot (\mathbf{v} - \mathbf{v}_\Sigma) + D_c \nabla c \mu]\!] \,. && \text{(SI.10)} \end{aligned}$$

The reduced dissipation inequalities (SI.9) - (SI.10) lead to the standard definition of the volumetric Cauchy stress tensor and the chemical potential:

$$\sigma^\alpha = \rho^\alpha \mathbf{F}^\alpha \frac{\partial \Psi^\alpha}{\partial \mathbf{F}^\alpha}\,, \quad \sigma_\Sigma = \rho_\Sigma \mathbf{F}_\Sigma \frac{\partial \Psi_\Sigma}{\partial \mathbf{F}_\Sigma}\,, \quad \mu^\alpha = \rho^\alpha \frac{\partial \Psi^\alpha}{\partial c^\alpha}\,. \tag{SI.11}$$

2 Numerical Simulations

In the following, we report further details on the numerical simulations presented in the paper, in order to expand the discussion about the effects of the principal parameters of the model on the network dynamics. For each snapshot in the Figures 1-3, we report the morphology of the vessel network at the time at which the longest branch has reached a given distance from the initial capillary wall. At the bottom of each snapshot we specify the dimensionless incremental time from the formation of the initial sprout, in order to compare the dynamics of the branch formation without considering the time required for the initial sprout formation.

From Fig. 1, it is possible to observe that an increasing η will lead to a faster development of branches, even though the time required for the initial sprout formation does not change. On the other hand, the parameters λ (see Fig. 2) and δ (see Fig. 3) not only affect the time required for branching (with higher velocity of network formation for higher values of either λ or δ), but they also influence the dimensionless time at which the initial sprout forms. In particular, the time

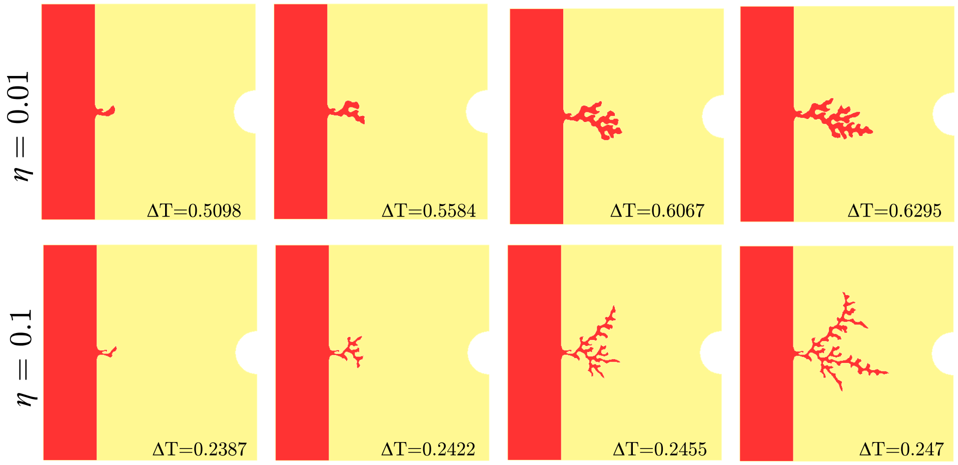

Figure 1: Morphological diagram of the simulated capillary morphology at different instant of time for different values of the parameter η, $\lambda = 1$, setting $\delta = 100$, $\xi = 5$ in a domain with dimensionless length and height equal to 0.32 and the interface initially placed in $x_\Sigma = 0.08$. At the bottom of each snapshot, we report the difference between the time at which the simulation was taken and the time of formation of the initial sprout, which is $T = 2.64373$ independently on the η. For each snapshot in the figures, we report the morphology of the vessel network at the time at which the longest branch has reached the same distance from the initial capillary wall.

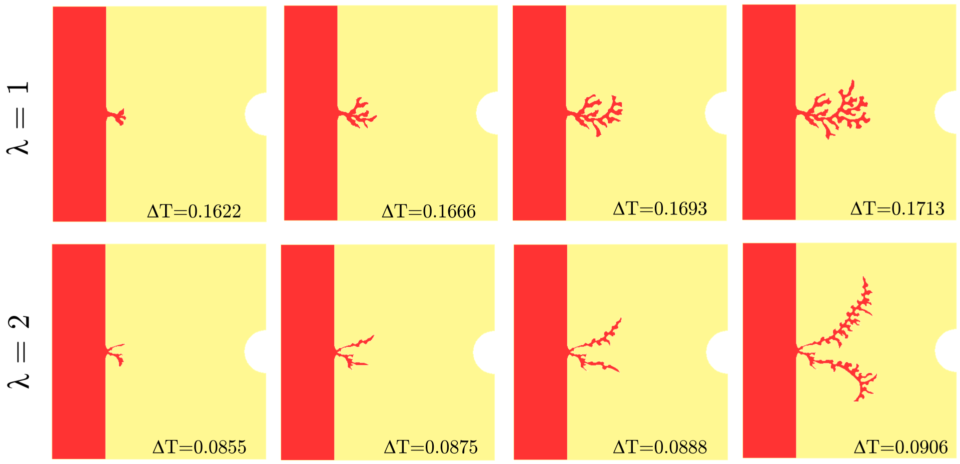

Figure 2: Morphological diagram of the simulated capillary morphology at different instant of time for different values of the parameter λ, setting $\delta = 100$, $\eta = 0.05$, $\xi = 10$ in a domain with dimensionless length and height equal to 0.32 and the interface initially placed in $x_\Sigma = 0.08$. At the bottom of each snapshot, we report the difference between the time at which the simulation was taken and the time of formation of the initial sprout, which is $T = 1.3219$ and $T = 0.6609$ for $\lambda = 1$ and $\lambda = 2$, respectively. For each snapshot in the figures, we report the morphology of the vessel network at the time at which the longest branch has reached the same distance from the initial capillary wall.

required for the initial sprout almost double whilst doubling the value of λ, whereas the dependency on δ is nonlinear. In all cases, the branching velocity increases as the capillaries approach the tumour.

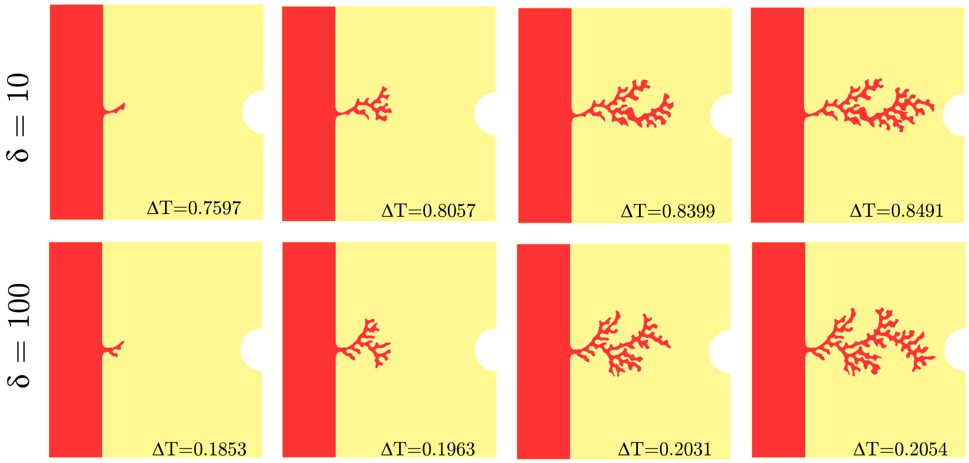

Figure 3: Morphological diagram of the simulated capillary morphology at different instant of time for different values of the parameter δ, setting $\lambda = 1$, $\eta = 0.032$, $\xi = 10$ in a domain with dimensionless length and height equal to 0.32 and the interface initially placed in $x_\Sigma = 0.08$. At the bottom of each snapshot, we report the difference between the time at which the simulation was taken and the time of formation of the initial sprout, which is $T = 3.2918$ and $T = 1.3218$ for $\delta = 10$ and $\delta = 100$ respectively. For each snapshot in the figures, we report the morphology of the vessel network at the time at which the longest branch has reached the same distance from the initial capillary wall.

Finally, we remind that the reported dimensionless time can give an indication of the speed of the different phases of the process once that the diffusion coefficient of the VEGF and its decay in the healthy region are known. In particular, considering a diffusion coefficient of $10\,\mu\mathrm{m}^2/\mathrm{s}$ [1] and a decay rate of the VEGF of $0.456\,\mathrm{h}^{-1}$ [2], the characteristic velocity is equal to $3.055\,\mathrm{mm/day}$. Thus, computing the average velocities for the depicted snapshots, it is possible to calculate an initial velocity of about $\sim 0.18 - 0.2\,\mathrm{mm/day}$ and $\sim 1.8 - 2\,\mathrm{mm/day}$ setting $\eta = 0.01$ (see Fig. 1) and $\delta = 10$ (see Fig. 3), respectively. Such values are comparable to the ones reported in literature [3, 4]. In all cases, the velocity of the capillary formation is higher when the new vessels approach the tumor. Nonetheless, we remark that in the present work we consider only one initial sprout, whereas the data on the velocities are extrapolated from experiments in which up to 30 sprouts initially develop [3]. Furthermore the simulations have been obtained considering a fixed concentration of VEGF at the tumor boundary, whereas a time-dependent conditions at the tumor interface can be useful to consider the physiological feedback between the tumor oxygenation due to the onset of the new vasculature and the secretion of VEGF in response to hypoxic condition. Both factors might explain the slight difference between the velocities predicted by the simulations and the ones reported in literature at later stages.

References

[1] Serini, G. *et al.* Modeling the early stages of vascular network assembly. *EMBO J.* **22**(8), 771–1779 (2003).

[2] Plank, M. J., Sleeman, B. D. & Jones, P. F. A Mathematical Model of an In Vitro Experiment to Investigate Endothelial Cell Migration. *Journal of Theoretical Medicine.* **4** (4), 251–270 (2002).

[3] Brem, H. & Folkman, J. Inhibition of tumor angiogenesis mediated by cartilage. *J Exp Med.* **141**(2), 427–439 (1975).

[4] Levine, H. A., Pamuk, S., Sleeman, B. & Nilsen-Hamilton, M. Mathematical modeling of capillary formation and development in tumor angiogenesis: Penetration into the stroma. *Bulletin of Mathematical Biology.* **63**, 801–863 (2001).
